# Aspects on the history of transmission and favor of distribution of viruses by iatrogenic action: perhaps an example of a paradigm of the worldwide spread of HIV

**DOI:** 10.1007/s00430-017-0505-2

**Published:** 2017-04-22

**Authors:** Lutz G. Gürtler, Josef Eberle

**Affiliations:** Max von Pettenkofer Institute, National Reference Centre for Retroviruses, Ludwig-Maximilians-University of München, Pettenkofer Str 9A, 80336 Munich, Germany

**Keywords:** Hepatitis B virus, Hepatitis C virus, Human immunodeficiency virus, Iatrogenic transmission, Slave trade, Vaccination

## Abstract

Transmission of infectious agents might be associated with iatrogenic actions of charitable help in health care. An example is the vaccination against yellow fever in USA that transmitted hepatitis B virus. Another example is injections of praziquantel for treatment and cure of schistosomiasis in Central and Northern Africa, with a focus in Egypt that has spread hepatitis C virus. There is no indication that human T-lymphotropic virus type 1 was spread by injection treatment for African trypanosomiasis, syphilis and treponematosis, but these treatments might have contributed to the early spread of human immunodeficiency virus type 1 (HIV-1) in Central Africa. Slave trade contributed as well to the spread of viruses from Africa to the Americas; it was stopped in 1850. Until that date HIV-1 was not transported to the Americas. By analysis of nucleic acid sequence data it can be concluded that the continental spread of HCV and HIV-1 might have started around 1920 with an exponential phase from 1940 to 1970. Further iatrogenic actions that promoted the spread of HCV and HIV-1 might be vaccinations to prevent deadly diseases. The successful vaccination was followed by diminution of the infectious agent in the population such as small pox, yellow fever and measles. Measurements to reduce the spread of plague and cholera were further benefits increasing survival of diseased subjects in a population. Thus, the reduction of exposure to deadly infectious agents might have given a chance to HIV-1 infected subjects to survive and for HIV-1 to be distributed around the world starting from Central Africa in the 1950s.

## Introduction

Caring for ill and injured members of a community is as old as mankind. Specialization in health care started in archaic communities with certain skill and experience and finally iatrogenic action by assisting to reach an improved outcome of a disease or of pregnancy. Iatrogenic bacterial transmission to delivering women and its reduction by hand disinfection was nicely demonstrated by Ignaz Semmelweis in 1847 [1]. Per definition iatrogenic transmission of infectious agents may be caused by persons as medical doctors, paramedical staff, midwives up to finally traditional healers. While transmission of virus, such as influenza virus, by respiratory secretions was well known since thousands of years knowledge of transmission of virus by blood was not realized, and therefore, not important. The extended risk of virus transmission by blood started only in the early 1920s when injection of drugs for treatment of diseases as schistosomiasis and syphilis was introduced, in the so-called injection century [2].

Blood transfusion is a further possibility of spreading viruses. The first efforts of improving anaemia by blood transfusion started around 1901 with the identification of blood groups by Landsteiner and the use of different salts as anticoagulant in 1915 by Lewisohn [3]. At that time presence of virus in blood had been published: in 1881 Carlos Findlay identified the mosquito transmitted cycle of yellow fever and in 1900 Walter Reed used blood of yellow fever diseased to inoculate this virus to human volunteers [4].

In this review a few examples of the history of iatrogenic transmission of viruses are given, discussing also the aspect of time delay until scientific results may have reached the medical community. It has to be stated as well that all the injection activities had been performed under the aspect of noble goals to treat and cure infectious disease [5]. In a retrospective aspect still at that time the implementation of rules of hygiene in medicine, initiated in 1847 by Semmelweis and 1867 by Joseph Lister, had a long delay to be accepted.

## Hepatitis B virus (HBV) transmission linked to yellow fever virus (YFV) vaccination

Approx. 300,000 US American soldiers were vaccinated in 1942 against YFV before being send to East-Asia with 3 different vaccine preparations [6, 7]: two of the vaccine lots were stabilized with human serum from volunteers; the third lot was serum free. Approx. 50,000 vaccinees developed jaundice (group I) 14–15 weeks after vaccination, and were hospitalized in army hospitals, while the others who received another serum stabilized vaccine (group II) and the serum-free vaccine (group III) remained anicteric. Around 40 years later between 170 and 220 of those vaccinees could be traced and their hepatitis B virus (HBV) infection markers analyzed. Members of the icteric group showed the following parameter profile: HBsAg positive: 0.5% (1/216); anti-HBs and anti-HBc positive: 90% (199/216); anti-HBc positive: 7% (16/216) and anti-HAV positive: 75% (165/216). Members of the exposed group were: anti-HBs and anti-HBc positive: 70% (120/171); anti-HBc positive: 6% (10/171) and anti-HAV positive: 73% (124/171). Members of the ‘control’ group, who received serum-free vaccine, were: anti-HBs and anti-HBc positive: 6% (13/205); anti-HBc positive: 7% (14/205) and anti HAV positive: 63% (129/205).

The anti-HAV infection rate corresponds to the prevalence value of a Western population at the time during World War Two. The anti-HBc titer of 6 plus 7% in the ‘control’ population corresponds as well to the HBV prevalence and natural course of the disease in the general population at that time [8], while the high anti-HBs titer prevalence of 90% in the symptomatic and 70% in the exposed group is a clear indicator that HBV was transmitted by the YFV vaccine. The HBV genotype from the one person still being HBsAg positive was characterized as genotype A2 (adw2) [6, 9], which is still a common genotype in the US American population [10].

YFV vaccine preparation started in 1928, and data of the circulation of 2 different agents causing hepatitis was reported as early as 1885, with the characteristics of a so-called epidemic catarrhal jaundice that was affecting mainly children that was published in 1914 [9]. Thus, the iatrogenic transmission of HBV by YFV vaccination could have been avoided bundling all the available information in 1942 [7].

## Hepatitis C virus (HCV) transmission by injection of drugs to treat disease

HCV is generally only transmitted by blood and not as HBV by sexual activity and blood, and therefore, HCV has been transmitted in historic times by scarification, tattooing, circumcision etc. [11]. Age of evolution of HBV was calculated with ca 30,000 years, that of HCV between 5000 and 20,000 years [12]. The country with the highest HCV prevalence in the world is Egypt with an average prevalence of 14.7% [13–15], that is higher in people aged >50 (23%) and lower in people aged <35 years (8.5%) [13, 16]. Anti-schistosomial therapy campaigns were performed from 1960 to 1970 by parenteral injection of praziquantel and were the leading risk factor for the forced HCV transmission during that time. Logistic regression analysis correlated a high HCV prevalence with increasing age, bilharziosis, praziquantel injection and residence close to the Nile or in the Nile-delta [11, 15]. The high HCV prevalence was followed by an increasing number of patients with liver cirrhosis and hepatocellular carcinoma [16, 17]. When the hygienic deficits causing HCV transmission were analyzed several factors were identified: reuse of syringes and needles, problems with sterilization, rare hand washing and untrained personnel [14]. Among the Egyptian HCV infected patients ca 86% were infected with HCV genotype 4 indicating a unique way of distribution [18], compared to Iraq with 60% and Syria with 57%, while in Turkey and Cyprus genotype 1 was most prevalent with 82 and 68%, respectively [18].

A small area with a high prevalence of HCV genotype 5a that was most probably transmitted by iatrogenic action was observed on Rhodes island. While in Greece HCV-5 has a prevalence of 1.9% (19/973), in Rhodes 13% (16/122) were found; 82% (13/16) of the patients infected with HCV-5a in Rhodes were females and the average age was 62 ± 6 years [19]. The iatrogenic route of genotype 5a, that is usually prevalent in Southern Africa, could fairly be linked to dental or surgical interventions.

A further possible event of iatrogenic transmission of HCV-1b was reported in 2002 from the small town of Camporeale in Sicily. The general HCV prevalence was 10.4% (75/721), while in people >60 the prevalence was 34% (48/141) and in subjects 10–29 years it was 0.4% (1/225); correlation analysis showed that a previous hospitalization was highly linked with HCV infection [20].

### Spread of HCV in the Congo Basin in Central Africa by iatrogenic action

Several studies were performed in Central Africa to determine the HCV genotype distribution in various cohorts and countries and to calculate the time of rapid spread of HCV transmission [21–24]. In Congo-Kinshasa a cohort of 299 males was investigated, 13.7% (41/299) of the patients were seropositive and 3.7% (11/299) viraemic; age correlation revealed that 13.7% (10/73) of those older 50, and 0.5% (1/211) younger 50 years were viraemic. The HCV genotypes were 4c, 4k, 4l and 4r and thus it was concluded that different separate HCV epidemics by parenteral injections, iatrogenic or another unidentified routes in the mid-twentieth century caused this epidemic [19, 21]. In a more recent study of 839 participants 26% (217/839) were anti-HCV positive and genotype distribution of 4k and 4r was detected in hospital A, with a risk factor of intramuscular and intravenous injection therapy for tuberculosis, while genotype 4k in hospital B in Kinshasa was associated with injection of anti-malarias. An independent risk factor for HCV infection was injection before 1960 and injections at a venerology clinic [22]. Iatrogenic transmission of HCV in the mid-20th century was the most probable conclusion [22].

A study performed in the Central African Republic in 905 subjects aged >55 on the molecular epidemiology of HCV revealed a high prevalence of HCV-4 (82.8%, 48/58), with subtypes 4k, 4c and 4f [23]. Phylogenetic analysis of the NS5B gene nucleic acid sequences allowed to calculate the age of the most recent common ancestor (MRCA) as the year 1539 and the Bayesian skyline plot indicated a rapid increase of the prevalence from 1930 to 1980. It was hypothesized that iatrogenic activity caused this increase [23]. When phylogeography and history of HCV in Gabon were evaluated it was found that 11% (455/4042) of the samples were seropositive. HCV-4 was prevalent in 92%, and subtype 4e dominated with 57.3% [24]. The HCV prevalence in the 9 provinces varied from 3.7 to 20.8%. A history of parenteral injection, hospital admission and age over 55 years were independently significant risk factors for HCV infection. Different diseases treated in the single provinces and different implementation of vaccination programs might have contributed to the divergent HCV prevalence in the 9 provinces. As discussed above for the other Central African countries iatrogenic transmission in the first part of the 20th century is the most probable route of HCV transmission also in Gabon [24].

Cameroon is a further Central African country with a high HCV prevalence [25] and with an available medical history of treatment records of trypanosomiasis, treponematosis, syphilis and leprosy [5]. In southern Cameroon 451 inhabitants were investigated and 56% (252/451) were found to be anti-HCV positive; HCV genotypes were analyzed in 171 specimens: 18.1% (31/171) of those were genotype 1, 27.5% (47/171) genotype 2 and 54.4% (93/171) genotype 4 [25]. Risk factors of being HCV infected were age >60 years, being vaccinated against small pox virus, having received >4 injections and traditional male circumcision, while the medical male circumcision showed no correlation. Calculation by the Bayesian skyline plot indicated the time of accelerated spread of all 3 HCV genotypes between 1920 and 1960 [23, 25]. Iatrogenic transmission was also in Cameroon an essential promoter of HCV spread at that time.

### Spread of HCV linked to slave trade

Looking at further ways of HCV transmission the transport (migration) of people being chronically infected with HCV is a possibility. A study performed at Amsterdam in 189 patients revealed in 178 the presence of HCV-2, determined by nucleic acid sequencing. HCV-2 could be subdivided in 8 distinct subtypes [26]. By analyzing the demographic information of the infected subjects, the phylogenetic age of the HCV-2 strains and the molecular clock of mutations, the conclusion was drawn that HCV-2 was transferred to Amsterdam by transatlantic slave trade during colonial time around 1700–1850. One part of the HCV strains was introduced by bidirectional transport of slaves from West-Africa (Ghana/Benin), and the other part from Indonesia and Javanese workers to and from Surinam [26].

In a further study on HCV nucleic acid sequences performed in Guinea-Bissau the origin of HCV-2 was calculated to have evolved in this region around 1470. By virus migration in slaves HCV-2a and HCV-2c were transported repeatedly to Madagascar and Martinique [27]. Thus, in conclusion slave trade was an effective vehicle of spreading chronic persistent viruses, between continents while iatrogenic transmission was linked to regional spread [26, 27].

## Human T-lymphotropic virus (HTLV-1 and HTLV-2) transmission by iatrogenic action

In contrast to HBV and HCV the retrovirus HTLV is a strictly cell bound virus that is usually absent from serum and is transmitted by whole blood, as blood transfusion, and by sexual contact. HTLV is an old virus that circulates in monkeys since ca 50,000 and in man since ca 25,000 years [28]. HTLV-1 and -2 are distributed worldwide [29], while HTLV-3 and -4 are rare. HTLV-5 might not be a new species but a divergent STLV-1 [30]. All HTLV-3 and -4 cases reported were found in Central Africa and mainly transmitted by monkey bite or lesion contaminated by monkey blood and then by sexual contact [31–33]. In African hunters monkey bite is still frequent [33].

Iatrogenic transmission of HTLV-1, together with HCV-4, in colonial Equatorial Africa by injection of pentamidine for treatment of sleeping sickness from 1936 to 1953 was reported in a cross-sectional survey in 2010 [34]. The age related profile gave clear evidence for transmission of HTLV-1 and HCV during this period.

As discussed within the HCV topic also slave trade was a way of spreading HTLV-1, which could be followed up by analyzing the different subtypes A to C. Subtype A was found in the Caribbean area, South America and in the Ainu population in Japan, subtype B in Japan and India, and subtype C in West-Africa and the Caribbean area [35]. The West African subtype C was most probably spread by slave trade, while the East Asian strains migrated with monkeys and humans via the Bering Strait to the Americas [36].

In conclusion HTLV-1 is a further virus causing chronic disease that was spread by iatrogenic treatment during colonial time, but to a lower extent than HBV an HCV.

## HIV-1 spread and the possible contribution of iatrogenic transmission

HIV-1 is a retrovirus causing chronic infection that is transmitted by cells and plasma of blood and sexual contact. According to molecular clock analysis HIV-1 with its 4 groups had been present in Africa in man around 1890–1930 or earlier and in chimpanzees before 1460 dependent on the viral gene analyzed [37, 38]. The human HIV-1 is a recombinant monkey virus, SIVcpz, transmitted from the chimpanzee (*Pan troglodytes troglodytes*) [39], and HIV-2 a virus transmitted from sooty mangabey monkey (*Cercopithecus atys*) that was spread according to the calculation of the molecular clock around 1933–1945 to man [38]. The oldest HIV-1 isolates had been found in preserved samples from 1959 and 1960 in Kinshasa [37] and showed with subtypes A and D a high sequence divergence suggesting a period of subtype evolution in man, possibly for 40 years, or two independent transmission events of two divergent SIVcpz [40]. According to the virus groups found in humans there had been at least 4 different zoonotic transmissions of HIV-1, associated with the transfer of group M to P, and at least 7 transmissions of HIV-2. Chimpanzee viruses (SIVcpz) corresponding to group O and P were transmitted as well to gorillas in some areas of Cameroon [41].

Spread of HIV in man: the speed of spread of HIV-1 subtypes A and D from Central Africa to East Africa was low before 1950 but grew exponentially during the 1970s [40, 42]. A possible reason for the accelerated spread might be industrial evolution, associated with a higher frequency of use of syringes and needles as discussed above [2]. Further reasons might be the adaptation of HIV-1 to the HLA-C*15, HLA*A02 and APOBEC3 genotypes [43, 44], and the prevalence and driving force of tuberculosis [45]. The risk of HIV-1 transmission by parenteral iatrogenic procedures and sexual exposure is very high during the early stage of HIV-1 infection due to the very high viral load and might have been a further reason for the exponential spread [46]. Looking at the time line and geography of HIV-1 appearance all available data indicate that HIV-1 emerged in Central Africa (Congo basin) and its exponential spread started around 50 years ago [37].

When slave trade is considered there is, as discussed, enough evidence of transport of HBV, HCV and HTLV-1, however, HIV-1 was not transferred to the Americas; either subjects infected with HIV-1 died during the ship transport, or HIV-1 had not entered humans up to 1850 when slavery was stopped in the West, Central and East African populations [47]. HIV-2 emerged in West Africa around 1940 [37] and its spread is mainly linked to human migration and behavior [46–49] and fairly to iatrogenic transmission.

## Further possible reasons favoring HIV spread some decades ago

- Influence of human superspreaders on the distribution of HIV-1: the US American HIV-1 epidemic started around 1970, it was preceded by a Caribbean epidemic in 1963 and one patient (patient zero) was discussed of having triggered the AIDS epidemic [50, 51]. The phylogenetic tree of nucleic acid sequence analysis of the few isolates from that time in USA indicated a wide heterogeneity of the subtype B strains and neither biological nor historical evidence was found that one patient was the source or index case of spread of HIV-1 subtype B in North America [50, 51]. Considering the worldwide distribution and spread of HIV with its 2 types, 11 groups and >25 subtypes the contribution of spread by the sexual activity of a single person or few persons is irrelevant.
- Influence of the availability of syringes and anticoagulant allowing the injection of blood: a further possibility of the emergence of HIV-1 is the injection of monkey (chimpanzee) blood by traditional healers that was performed in the area of the great lakes of Central Africa [52].
- Influence of the pathogenicity of HIV: the change of the pathogenicity of the chimpanzee virus, SIVcpz, after transfer to man from very aggressive, killing people in a few weeks, to low, allowing a chronic course for a decade, would be a further reason for the onset of the epidemic some 100 years ago.
- Influence by the success of vaccination and disease treatment: a further possibility of iatrogenic contribution would be as well the diminution of deadly viruses in the Central African populations by vaccination against small pox virus between 1850 and 1950 [53], yellow fever virus since 1945 [5] and measles virus since ca 1970 [54–56]. Besides vaccination [57] further iatrogenic actions helping to survive diseased people with impaired immunity would be the successful treatment of sleeping sickness followed by reduction of spread of *Trypanosoma brucei*, or similarly treatment of plague, cholera, malaria, etc. Absence of those infectious agents that kill patients with severe immunodeficiency would have contributed to preserve the life of HIV-1 infected subjects with minor immunodeficiency and thus allowing the spread of HIV by the sexual route.

## Conclusion

Several possibilities have to be discussed for the worldwide burst of HBV, HCV and HIV-1 and their evident spread in the 1940–1970s. The main possibility is lack of hygiene during mass vaccination and mass injection treatment of diseases as for African trypanosomiasis and syphilis. Due to poor hygienic conditions by persons performing those actions and due to the absence of knowledge of the presence of blood borne viruses at that time HIV-1 transmission was promoted. A further possibility is that successful drug treatment of tropical diseases and vaccination supported a longer survival of HIV infected subjects allowing them to spread the virus within their community.

The occurrence of HIV is a reason for health workers caring for patients to actualize knowledge on infectious agents and their transmission route, to question and check the validity of recommendations of action periodically and to balance beneficial and harmful events in sick people for years.
